# No association between subclinical hypothyroidism and dyslipidemia in children and adolescents

**DOI:** 10.1186/s12887-020-02318-z

**Published:** 2020-09-16

**Authors:** Ashkan Habib, Asadollah Habib

**Affiliations:** 1 School of Medicine, Shiraz University of Medical Sciences, Shiraz, Iran; 2 Department of Endocrinology, School of Medicine, Kazerun Branch, Islamic Azad University, PO Box: 71384-37984, First Floor, Zafar Building, Zand St, Shiraz, Iran

**Keywords:** Lipid, Children, Thyroid, Hypothyroidism

## Abstract

**Background:**

There are controversies about the correlation between higher levels of thyroid stimulating hormone (TSH) and dyslipidemia in children. This study was designed to assess the relation between lipid profile components and TSH levels in children.

**Method:**

This cross-sectional study was performed in a pediatric endocrinology growth assessment clinic in Shiraz, southern Iran. Children aged 2–18 years who referred to the clinic from January until April 2018 were included. TSH levels equal or above 5 mIU/L and lower than 10 mIU/L with normal free T4 (FT4) were considered as having subclinical hypothyroidism (SH).

**Results:**

Six hundred sixty-six children were euthyroid while 181 had SH. No significant difference was found between the mean serum total cholesterol (*P* = 0.713), LDL-C (*P* = 0.369), HDL-C (*P* = 0.211), non-HDL-C (*P* = 0.929), and triglyceride (*P* = 0.215) levels between euthyroid children and subjects with SH. There was also no significant difference in the prevalence of dyslipidemias in any lipid profile components between the two groups. The adjusted correlation was not significant between TSH levels and any lipid profile component.

**Conclusion:**

Based on the results of our study, we found no correlation between SH and dyslipidemia in children. The association between dyslipidemia and SH in children still seems to be inconsistent based on the results of this and previous studies. We recommend a meta-analysis or a significantly larger retrospective study on this subject.

## Introduction

Subclinical hypothyroidism (SH) is defined as elevated thyroid stimulating hormone (TSH) levels, while T4 or free T4 (FT4) levels are normal [1]. It is a common disorder with a prevalence of 4–15% in the adult community [2, 3], while in the pediatric population, its prevalence is slightly lower than 2% [2, 4].

Although initially symptom free, 2–5% of SH patient progress to overt hypothyroidism each year [5]. In addition, SH has been linked to several complications such as decreased cardiac output and higher chances of cardiac diseases [6–12], neuromuscular and neurobehavioral alterations, insulin resistance [8, 13–16] and specifically, obesity. Several studies have linked SH to higher BMI and BMI Z-scores [17–19], although weight reduction plans have resulted in normalization of TSH concentrations in some cases [20, 21].

Association of SH and dyslipidemia has been less clear cut. Studies have correlated SH in adults with higher levels of total cholesterol, low-density lipoprotein (LDL), non-high density lipoprotein (HDL), triglyceride (TG), and lower levels of HDL [22–24]. However, in a study by Meisinger and colleagues, only a higher TG level was correlated with higher levels of TSH in male participants. Higher levels of total cholesterol and LDL-C were only found in female participants [25].

In children however, reports about the correlation between the higher levels of TSH and dyslipidemia have been controversial. Some studies have shown higher levels of TC, LDL, and TG with an increase in TSH [26–30]. Conversely, another study showed that only higher levels of TG was positively correlated with an increase in TSH level [31].

As there are controversies about the association of dyslipidemia and SH in children, we designed this cross-sectional study to compare lipid profiles, including TG, total cholesterol, LDL-C, and HDL-C in children with SH compared with children with the euthyroid state.

## Method

### Study design

This was a cross-sectional study on children aged 2 to 18 years who referred to a pediatric endocrinology growth assessment clinic in the urban area of Shiraz city, southern Iran for routine growth follow-up from January until April 2018. The clinic is also a tertiary referral center where patients with other endocrine disorders are visited as well. Approximately eighty to ninety patients per day are visited in this center.

After obtaining informed consent from the parents, the children’s blood samples were tested in a single laboratory for serum total cholesterol, LDL-C, HDL-C, Non-HDL-C, TG, TSH, and FT4 levels simultaneously in a non-fasting state. The non-fasting state was chosen as a result of high non-compliance and questionable compliance of the parents, especially in 2-4-year-old subjects, although it should be noted that fasting is generally not required for determination of a lipid profile [32]. The weight and height of the subjects were measured using Seca scale (0.1 kg precision) and stadiometer (0.1 cm precision), respectively, while they were lightly dressed and were asked to take off their shoes.

### Inclusion criteria

For this study, inclusion criteria were: 1- age of 2–18 years; 2- the presence of normal free T4 (0.8–1.8 ng/dL); and 3- TSH level between 0.3 and < 10 mIU/L as recommended by the 2014 European Thyroid Association guideline on the management of SH in children [33].

### Exclusion criteria

Exclusion criteria were 1- children who were on levothyroxine therapy at the time of assessment; 2- ongoing use of medications that might interfere with thyroid function test or lipid profile such as anti-thyroid medications, corticosteroids, and thiazides; 3- children with familial hyperlipidemia; and 4- diseases that might affect lipid profile such as diabetes mellitus (DM), kidney disease, rheumatologic diseases, and other endocrine diseases.

### Anthropometric data

847 children, including 366 (43.2%) boys and 481 (56.8%) girls, had the inclusion criteria and were selected for this study. For the precise calculation of body mass index (BMI), body mass index standard deviation score (BMI-SDS) and BMI percentile, UptoDate calculators, which are based on the growth charts of the Center for Disease Control (CDC), were used.

### Measurement of serum lipid profiles and thyroid functions

Serum TSH and lipid profile were measured using Cobas e411 Analyzer (Mannheim, Germany) with electrochemiluminescence immunoassay (ECLIA) method and Dirui CS- T240 Auto chemistry Analyzer (Changchun, China) using Pars Azmoon kits (Iran), respectively. Assay performance was controlled using Elecsys PreciControl Universal for serum thyroid profile and TrueLab N and TruLab P for lipid profile. Auto Analyser was calibrated using Elecsys TSH CalSet and TruCal U. Inter-assay coefficients of variation (CVs) for TSH are 1.56% for 1.37 mIU/L and 0.08% for 8.62 mIU/L, respectively while inter and intra-assay CVs were 3.2 mg/dL and 2.29% for total cholesterol and 1.6 mg/dL and 1.49% for triglyceride, respectively. The study was approved by the Research Institutional Review Committee of Islamic Azad University, Kazerun branch (reference number: 1398.125).

### Cut-off levels

Abnormal lipid profile cutoffs are based on the 2011 statement of the National Heart, Lung, and Blood Institute’s (NHLBI) Expert Panel on Integrated Guidelines for Cardiovascular Health and Risk Reduction in Children and Adolescents and the 2008 American Academy of Pediatrics’ (AAP) policy statement (Table 1) [34, 35].

**Table 1:** Definition of lipid levels in children from the 2011 Expert Panel Integrated Guidelines for Cardiovascular Health and Risk Reduction in Children and Adolescents

Category	Acceptable mg/dL (mmol/L)	Borderline mg/dL (mmol/L)	High
TC	< 170 (4.4)	170 to 199 (4.4 to 5.2)	≥ 200 (5.2)
LDL-C	< 110 (2.8)	110 to 129 (2.8 to 3.3)	≥ 130 (3.4)
Non-HDL-C	< 120 (3.1)	120 to 144 (3.1 to 3.7)	≥ 145 (3.8)
TG			
• 0 to 9 years	< 75 (0.8)	75 to 99 (0.8 to 1.1)	≥ 100 (1.1)
• 10 to 19 years	< 90 (1 mmol/L)	90 to 129 (1 to 1.5)	≥ 130 (1.5)
**Category**	**Acceptable**	**Borderline**	**Low**
HDL-C	> 45 (1.2)	40 to 45 (1 to 1.2)	< 40 (1)

Abbreviations: *TC* total cholesterol; *LDL-C* low-density lipoprotein cholesterol; *HDL-C* high-density lipoprotein cholesterol; *Non-HDL-C* non-high-density lipoprotein cholesterol; *TG* triglycerides

TSH levels equal to or above 5 mIU/L were considered abnormal. All participants with high TSH levels were considered for a second measurement. For these participants, second TSH levels were considered for the study.

### Study analysis

For analysis, the study group was divided into two age groups: 2–9 and 10–18 years, each representing before the start of puberty and after the start of puberty, respectively. Participants with TSH levels equal or above 5 mIU/L and lower than 10 mIU/L with normal free T4 levels were categorized as having SH. The relation between serum TSH and each lipid profile component (dependent variable) was evaluated using partial variable correlation, adjusted for age, sex, and BMI Z-score. Comparisons were performed using Student *t* test for continuous variables in Tables 2 and 3 and by Chi-square test for categorical variables in Tables 2 and 4. A *P* value < 0.05 was considered statistically significant in all comparisons with a 95% confidence interval. All statistical analyses were performed using SPSS software version 25.0 (SPSS, Chicago, IL, USA).

**Table 2:** Anthropometric characteristics of the study subjects

	Total (847)			Age 2–10 years (421)			Age 10–18 years (426)		
	Euthyroid (666)	Subclinical Hypothyroid (181)	P	Euthyroid (325)	Subclinical Hypothyroid (96)	P	Euthyroid (341)	Subclinical Hypothyroid (85)	P
Age	9.96 ± 3.40	9.98 ± 3.28	0.945	7.26 ± 2.047	7.54 ± 1.79	0.234	12.53 ± 2.25	12.74 ± 2.21	0.449
Sex (Male)	42.8%	44.8%	0.637	34.8%	39.6%	0.388	50.4%	50.6%	0.980
Height (cm)	135.20 ± 19.48	137.16 ± 18.37	0.226	121.17 ± 15.60	125.43 ± 14.11	0.017*	148.57 ± 11.97	150.40 ± 12.79	0.215
Weight (kg)	38.66 ± 20.22	41.31 ± 19.44	0.116	26.99 ± 12.23	31.54 ± 14.17	0.002*	49.79 ± 20.06	52.34±18.72	0.289
TSH (mIU/L)	2.52 ± 1.16	6.90 ± 1.52	< 0.001*	2.50 ± 1.15	6.80 ± 1.51	< 0.001*	2.53 ± 1.17	7.01 ± 1.52	< 0.001*
FT4 (ng/dL)	1.40 ± 0.25	1.43 ± 0.26	0.242	1.39 ±0.25	1.42±0.27	0.298	1.42 ± 0.25	1.44 ± 0.25	0.491
BMI Z score	0.16 ± 1.84	0.55 ± 1.80	0.012*	-0.08±1.92	0.49±1.95	0.011*	0.40 ± 1.73	0.62 ± 1.62	0.285
BMI	19.76 ± 6.14	20.98 ± 7.39	0.024*	17.50±4.68	19.19±5.44	0.007*	21.91 ± 6.57	23.00 ± 8.71	0.205

Abbreviations: *BMI* body mass index; *TSH* thyroid stimulating hormone; *FT4* free T4.

**Table 3:** Mean serum lipid profile components based on the subjects’ thyroid status

	Total (847)			Age 2–10 (421)			Age 10–18 (426)		
	Euthyroid (666)	Subclinical Hypothyroid (181)	P	Euthyroid (325)	Subclinical Hypothyroid (96)	P	Euthyroid (341)	Subclinical Hypothyroid (85)	P
TC (mg/dL)	160.50 ± 29.070	161.39 ± 28.694	0.713	161.66 ± 29.842	160.73 ± 26.664	0.782	159.38 ± 28.314	162.14 ± 30.971	0.431
LDL-c (mg/dL)	90.96 ± 24.996	89.10 ± 23.852	0.369	92.54 ± 25.514	88.06 ± 21.540	0.119	89.46 ± 24.436	90.27 ± 26.302	0.788
HDL-c (mg/dL)	47.94 ± 10.560	49.04 ± 10.361	0.211	48.62 ± 11.077	50.09 ± 10.910	0.253	47.29 ± 10.016	47.86 ± 9.630	0.637
Non-HDL-c (mg/dL)	112.56 ± 27.696	112.35 ± 28.136	0.929	113.04 ± 28.081	110.64 ± 27.286	0.459	112.09 ± 27.358	114.28 ± 29.107	0.515
TG (mg/dL)	104.98 ± 54.934	113.83 ± 91.342	0.215	97.18 ± 48.897	114.95 ± 104.861	0.111	112.40 ± 59.251	112.56 ± 73.757	0.983

Abbreviations: *TC* total cholesterol; *LDL-c* low-density lipoprotein cholesterol; *HDL-c* high-density lipoprotein cholesterol; *Non-HDL-c* non-high-density lipoprotein cholesterol; *TG* triglycerides

## Results

As shown in Table 2, of the 847 children in this study, 666 had TSH levels between 0.3 and 4.9 mIU/L who were considered as having euthyroid state while 181 children who had TSH levels of 5-9.9 mIU/L and were considered as having SH. There was no statistically significant difference in the mean age of the participants with euthyroid and SH states (9.96 ± 3.40 years vs. 9.98 ± 3.28, *P* = 0.945).

**Table 4:** Distribution of lipid profile abnormality based on thyroid status and age

	Total (847)			Age 2–10 (421)			Age 10–18 (426)		
	Euthyroid (689)	Subclinical Hypothyroid (184)	P	Euthyroid (338)	Subclinical Hypothyroid (100)	P	Euthyroid (351)	Subclinical Hypothyroid (84)	P
TC (mg/dL)									
Acceptable (< 170)	66.4%	65.7%	0.915	63.7%	65.6%	0.933	68.9%	65.9%	0.672
Borderline-high (170–199)	24.2%	23.8%		27.1%	26.0%		21.4%	21.2%	
High ( > = 200)	9.5%	10.5%		9.2%	8.3%		9.7%	12.9%	
LDL-c (mg/dL)									
Acceptable (< 110)	81.2%	83.4%	0.682	77.8%	85.4%	0.268	84.5%	81.2%	0.636
Borderline-high (110–129)	12.3%	9.9%		14.8%	9.4%		10.0%	10.6%	
High ( > = 130)	6.5%	6.6%		7.4%	5.2%		5.6%	8.2%	
HDL-c (mg/dL)									
Acceptable (> 45)	56.9%	61.9%	0.266	58.8%	63.5%	0.205	55.1%	60.0%	0.720
Borderline-low (40–45)	19.8%	20.4%		17.2%	20.8%		22.3%	20.0%	
Low (< 40)	23.3%	17.7%		24.0%	15.6%		22.6%	20.0%	
Non-HDL-c (mg/dL)									
Acceptable (< 120)	64.0%	62.4%	0.661	64.6%	63.5%	0.857	63.3%	61.2%	0.213
Borderline-high (120–144)	24.2%	23.2%		21.5%	24.0%		26.7%	22.4%	
High ( > = 145)	11.9%	14.4%		13.8%	12.5%		10.0%	16.5%	
TG (mg/dL)									
Acceptable (< 75/90)	41.0%	44.2%	0.522	38.2%	39.6%	0.967	43.7%	49.4%	0.400
Borderline-high (75–99/90–129)	26.7%	22.7%		23.7%	22.9%		29.6%	22.4%	
High ( > = 100/130)	32.3%	33.1%		38.2%	37.5%		26.7%	28.2%	

Abbreviations: *TC* total cholesterol; *LDL-c* low-density lipoprotein cholesterol; *HDL-c* high-density lipoprotein cholesterol; *Non-HDL-c* non-high-density lipoprotein cholesterol; *TG* triglycerides

42.8% of euthyroid children and 44.8% of the children with SH were male (*P* = 0.637). Overall, children with SH had higher BMI Z-scores than euthyroid children (*P* = 0.012); as a result, this parameter had to be adjusted when calculating the correlation (Table 2).

Table 3 shows the mean levels of lipid profile components in euthyroid children and children with SH and the subgroups of 2–10 and 10–18 years of age. There was no statistically significant difference in any of the lipid profile components between euthyroid children and children with SH and in the subsequent age-related subgroups (Table 3).

Table 4 shows the prevalence of dyslipidemia, in each of the lipid profile components, in euthyroid children and children with SH and their respective age groups. Overall, there was no statistically significant difference in the prevalence of any of the lipid profile dyslipidemias between the euthyroid children and children with SH and in the subsequent age-related subgroups (Table 4).

Table 5 shows the association between TSH levels and each of the lipid profile components based on the partial correlation method adjusted for age, sex, and BMI Z-score. No correlation was seen between TSH levels and any of the lipid profile components. The use of logistic regression was forgone due to the results of this study (Table 5).

**Table 5:** Correlation of lipid profile components with serum TSH levels

Lipid Profile components	Correlation coefficient (r)	*P* value
TC	0.033	0.331
LDL-c	0.015	0.657
HDL-c	0.039	0.257
Non-HDL-c	0.020	0.554
TG	0.019	0.584

Notes: Data are given in r and *P* values. Correlation coefficient was assessed by partial correlation method. Adjusted for age, sex, and BMI Z-score

Abbreviations: *TC* total cholesterol; *LDL-c* low-density lipoprotein cholesterol; *HDL-c* high-density lipoprotein cholesterol; *Non-HDL-c* non-high-density lipoprotein cholesterol; *TG* triglycerides

## Discussion

Relation of SH and dyslipidemia in children remains to be inconsistent as we found no correlation between TSH levels and lipid profile components. We also found no difference in the proportion of dyslipidemia and the mean serum lipid levels between euthyroid children and patients with SH.

Most past studies on this subject have found some aspect of dyslipidemia pertaining to SH although the results have been conflicting. In a study by Unal E and colleagues on 38 children with SH compared with a control group, SH led to increased dyslipidemia (increased TC and LDL) [26] and likewise, in a study by Witte and co-workers, it was shown that there was a significant positive association between TSH and all non-HDL parameters (total cholesterol, LDL-C, and TG) in children [27] and the latest study by Dahl and others on 228 children with SH showed that mild SH in children and adolescents was associated with higher rates of elevated total cholesterol and elevated non-HDL-c [30]. These studies have shown higher non-HDL dyslipidemia parameters in subclinical hypothyroid children while demonstrating no significant difference in HDL dyslipidemias between euthyroid and SH groups. Meanwhile, studies by Cerbone et al. and Paoli-Valeri et al. demonstrated the exact opposite. Cerbone and others showed that TG to HDL-C ratio (*P* = 0.01), and HDL-c were significantly lower (*P* = 0.003) in patients with SH compared with controls [28] and in the study by Paoli-Valeri and colleagues on 17 children with SH, the subjects with SH had significantly lower HDL-c levels [29]. These studies demonstrated no significant difference in total cholesterol and LDL between SH and euthyroid children.

A third group of studies, including ours, have shown no difference in total cholesterol, LDL-c, HDL-c or non-HDL-c between SH and euthyroid children. Çatlı et al., in a study on 27 children with SH compared with a control group, showed that there were similar serum lipoprotein levels and dyslipidemia frequency between the two groups [36] and the study by Nader and co-workers on 131 euthyroid children also found similar results, although a mild relation between TG and TSH was exhibited [31].

Further inspection of the studies for an explanation of the aforementioned differences did not yield results. We hypothesized several factors affecting the results including different dyslipidemia cut off levels, fasting state and the time span of subject inclusion. However none could clarify the differences. For example, while studies by Unal E and Nader NS were conducted in a fasting state, our study and Dahl AR were in a non-fasting state. However, these aforementioned studies had very different results. Another example is that our study and the studies by Paoli-Valeri et al. and Nader NS et al. were conducted on subjects from relatively short time spans while studies by Witte T and Dahl AR were on subjects from a longer time period. The studies on short time spans had different results while the ones on longer time periods were relatively similar. Regarding the different dyslipidemia cut offs, we noticed that despite the difference in normal lipid profile ranges of each study, the results were mostly obtained from differences in mean serum lipid levels, thus the different cut offs did not affect the primary results.

The question of whether SH can affect serum lipid levels is very important in preventing atherosclerosis in later years as studies have shown that atherosclerosis often begins in childhood with pediatric dyslipidemia a significant contributing factor [37]. Unlike SH, the association of overt hypothyroidism and dyslipidemia has been more consistent. Although levothyroxine (L-T4) treatment exerts some beneficial effects, there is no available data regarding the impact of therapy on metabolic outcomes in children with SH [28, 36]. The mechanism of the effect of thyroid hormones on lipid profile is not completely understood. However, thyroid hormones can reduce apoB lipoproteins via a non-LDL receptor pathway that leads to decreased liver apoB production [38]. It is also generally believed that thyroid hormones and their synthetic derivatives known as thyromimetics, can reduce serum cholesterol by their ability to increase LDL receptors, as demonstrated by a recent study illustrating that thyroid hormones and thyroid hormone receptor-β selective agonists, GC-1 and KB2115, were able to reduce serum cholesterol by inducing Cyp7a1 expression and stimulating the conversion and excretion of cholesterol as bile acids [39].

The advantage of our study over other studies is that a large number of children with SH were included, and also this was a cross-sectional study, while most of the other studies on the relation of TSH and serum lipid concentration are case-control studies spanning several years. A limitation of the study includes not recording serum Anti-TPO levels. Although serum Anti-TPO antibodies are not required for diagnosis of SH, they are recommended for considering therapeutic management of the disease and they would have certainly helped in better analysis of subjects. Precise subject puberty status was also not recorded.

## Conclusions

Based on the results of our study, we found no correlation between SH and dyslipidemia in children. The association between dyslipidemia and SH in children still seems to be inconsistent based on the results of this and previous studies. We recommend a meta-analysis or a significantly larger retrospective study on this subject.

## Data Availability

The datasets used during the current study are available through the corresponding author on reasonable request and upon permission from the university and the clinic.

## Abbreviations

TC: Total cholesterol
LDL-C: Low-density lipoprotein cholesterol
HDL-C: High-density lipoprotein cholesterol
Non-HDL-C: Non-high-density lipoprotein cholesterol
TG: Triglycerides
BMI: Body mass index
TSH: Thyroid stimulating hormone
FT4: Free T4
CI: Confidence interval
CDC: Center for disease control and prevention
